# Supplementary material for: A switch point in the molecular chaperone Hsp90 responding to client interaction
Source: Nat Commun. 2018 Apr 16;9:1472. doi: 10.1038/s41467-018-03946-x (PMC5902578; doi:10.1038/s41467-018-03946-x)
Supplement: Supplementary file 1 — Supplementary Information [file 41467_2018_3946_MOESM1_ESM.pdf]

A switch point in the molecular chaperone Hsp90 responding to client interaction

Rutz et al.

Supplementary Figures

Supplementary Figure 1

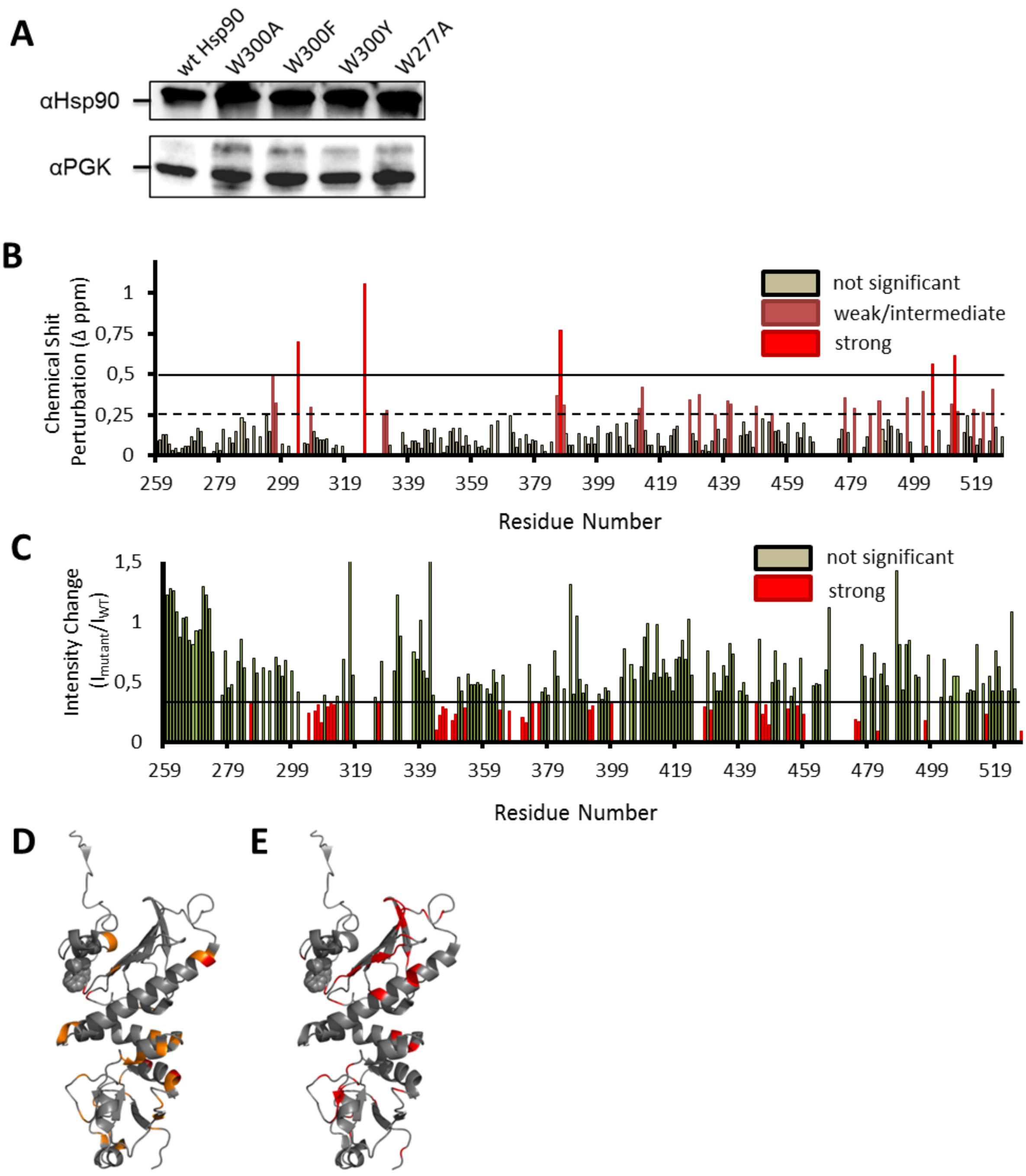

Supplementary Figure 1: Mutant expression levels, chemical shift perturbations (CSPs) and intensity changes; A) Expression levels of wt Hsp90 and viable W300/W277 mutants *in vivo.* Hsp90 levels in yeast were assessed by Western-Blot analysis using PGK (phosphoglycerate kinase) as a loading control. B) CSPs distributed over the Hsp90-M; CSPs were considered

significant above 0.25 ppm (dashed line) and weak/intermediate (orange) or above 0.5 ppm (solid line) as strong (red); C) Intensity changes distributed over the Hsp90-M; Intensity changes two standard deviations below (0.34) the normalized intensity (solid line) were considered as strong (red); D) CSPs mapped onto the Hsp90-M structure deduced form the crystal structure of the full-length closed Hsp90 (PDB ID 2CG9, residues 259-527); weak/intermediate CSPs in orange and strong CSPs in red; W300 is depicted as grey spheres E) Intensity changes mapped onto the Hsp90-M structure deduced form the crystal structure of the full-length closed Hsp90 (PDB ID 2CG9, residues 259-527); Strong intensity changes in red; W300 is depicted as grey spheres. A list of the affected residues can be found in Supplementary Table 1.

Supplementary Figure 2

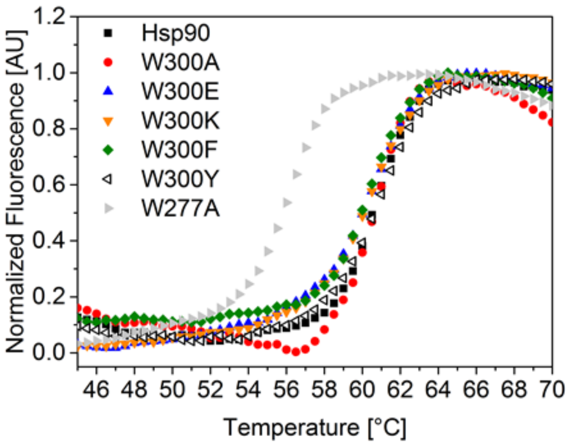

Supplementary Figure 2: Thermal stability of Hsp90 W300 mutants; TSA assay performed with different Hsp90 W300/W277 mutants. Melting curve for wt Hsp90 is shown in black, for W300A in red, for W300E in blue, for W300K in orange, for W300F in green, for W300Y in black bordered triangles and for W277A in grey. The measurements were performed in triplicates (n=3). Color code will be maintained for the mutants throughout the work. For melting temperatures see Table 1.

Supplementary Figure 3

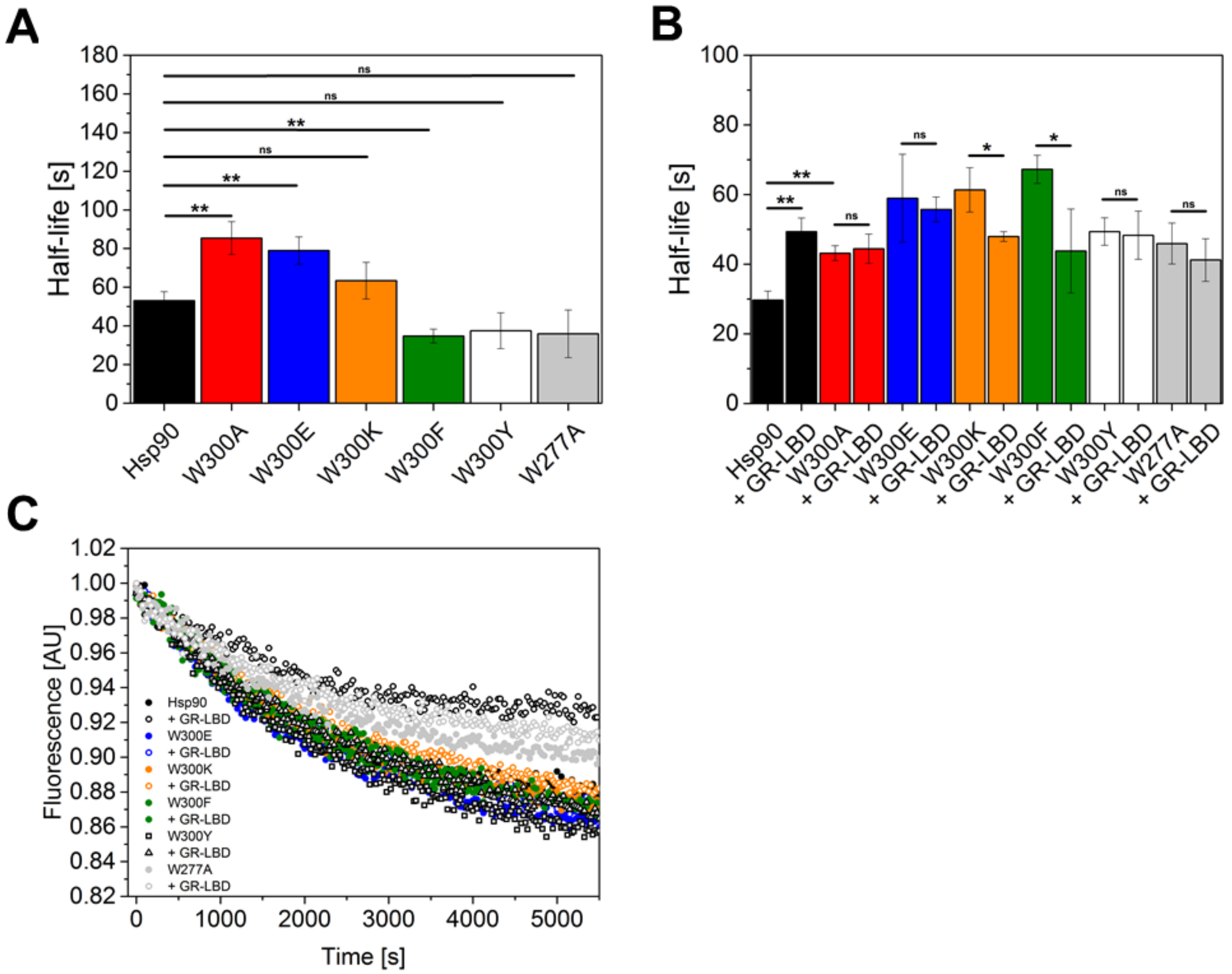

Supplementary Figure 3: The W300 mutations affect different Hsp90 conformations and the client interplay. A) Formation of the FRET hetero-dimer was monitored by mixing ATTO488 donor-labeled Hsp90/Hsp90 mutant with ATTO550 acceptor-labeled Hsp90/Hsp90 mutant. Mean reaction half-lives were derived from the original traces and plotted. The measurements were performed in triplicates (n=3). Black bars indicate standard deviations of three independent measurements. Statistical significance was assessed using a two-sample t-test and a level of significance of 0.05. B) Chase experiments with the open Hsp90 dimer in the absence and presence of GR-LBD. Chase experiments were started by the addition of wt Hsp90. Mean reaction half-lives were derived from the original traces and plotted. The measurements were performed in triplicates (n=3). Black bars indicate standard deviations of three independent measurements. Statistical significance was assessed using a two-sample t-test and a level of significance of 0.05. C) Chase experiments with the closed wt/W300E/K/F/Y and W277A Hsp90

dimer in the absence and presence of the GR-LBD. Chase experiments were performed in single-measurements (n=1) and initiated by the addition of wt Hsp90.

Supplementary Figure 4

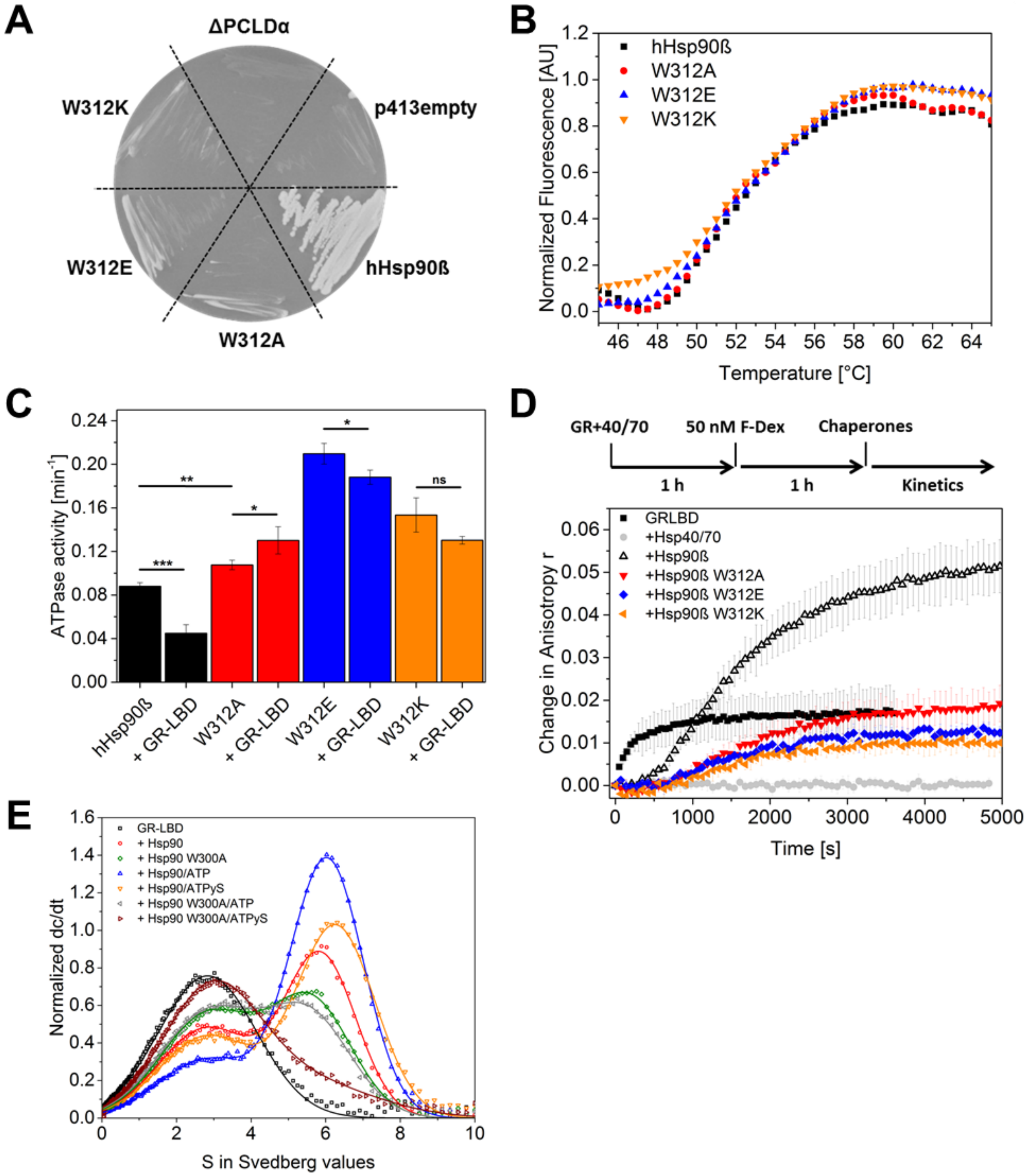

Supplementary Figure 4: The evolutionary conserved role of W300 in human Hsp90β; A) Viability of yeast containing human Hsp90β or W312 mutants as the sole source of Hsp90. Yeast were plated on selective media including 5`FOA. The shuffling strain ΔPCLDα and ΔPCLDα transformed with the empty p413-GPD vector were plated as negative controls.

ΔPCLDα transformed with p413-GPD containing wt human Hsp90β served as positive control. Mutations in human Hsp90ß are as indicated. B) TSA assay of human Hsp90β and human Hsp90β W312 mutants. The measurements were performed in triplicates (n=3). C) ATPase activity measurements of wt human Hsp90β and W312 mutants in the absence and the presence of the GR-LBD; Hsp90β is shown in black, W312A in red, W312E in blue and W312K in orange; Shown are mean ATPase activities of three independent measurements including the standard deviation represented as black bars (n=3). Statistical significance was assessed using a two-sample t-test and a level of significance of 0.05. D) Schematic representation of the hormone-binding recovery experiment. Binding kinetics of 50 nM F-DEX and GR-LBD in the absence of chaperones in black squares, in the presence of Ydj1, Hsp70 and 2 mM ATP in grey, in the presence of the full chaperone system and wt human Hsp90β in black open triangles, in the presence of the Hsp90β W312A in red triangles, Hsp90β W312E in blue rhombs and Hsp90β W312K in orange triangles. The measurements were performed in triplicates (n=3). Error bars indicate the standard deviations for three independent measurements. E) Exemplary AUC traces of ATTO488-labeled GR-LBD in black in the presence of wt Hsp90 (red trace) and Hsp90 W300A (green trace) without nucleotide and in the presence of 2 mM ATP (blue and orange traces) or ATPγS (grey and brown traces).

Supplementary Figure 5

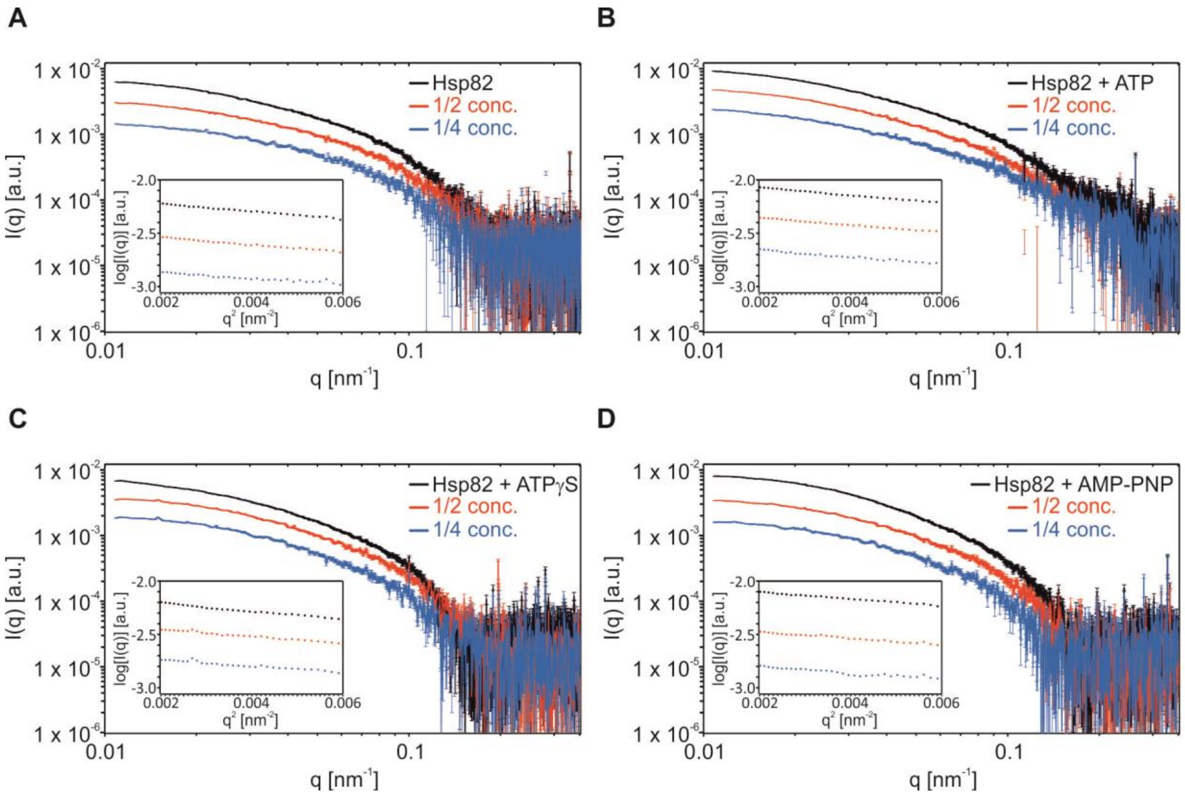

Supplementary Figure 5: SAXS scattering curves. Experimental X-ray scattering data of Hsp82, free (A) and in presence of ATP (B), ATPγS (C), or AMP-PNP (D) recorded at different sample concentrations in the range 1-5 mg/ml. Both the *q*, and *I(q)* axes are shown in a logarithmic representation. The angular ranges from 0.0012 - 0.4 nm^{-1} are compared.

Supplementary Figure 6

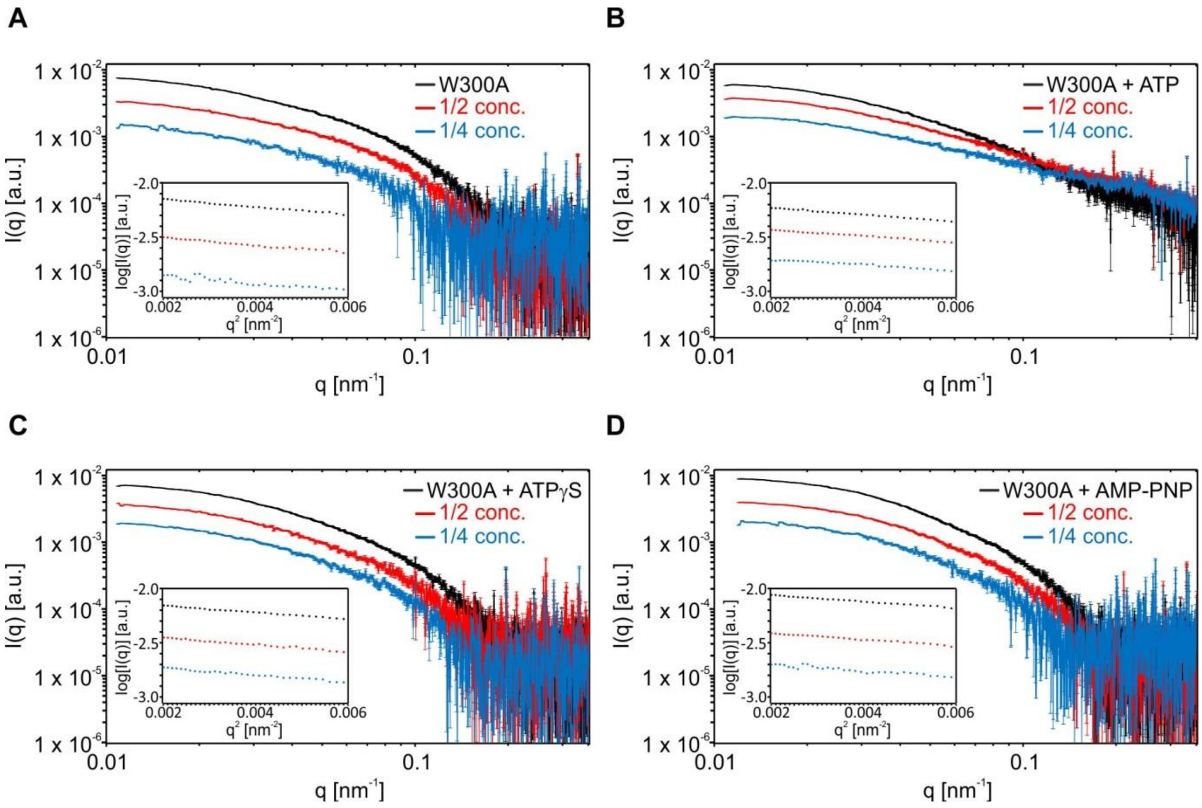

Supplementary Figure 6: SAXS scattering curves. Experimental X-ray scattering data of Hsp82 W300A, free (A) and in presence of ATP (B), ATPγS (C), or AMP-PNP (D) recorded at different sample concentrations in the range 1-5 mg/ml. Both the *q*, and *I(q)* axes are shown in a logarithmic representation. The angular ranges from 0.0012 - 0.4 nm^{-1} are compared.

Supplementary Figure 7

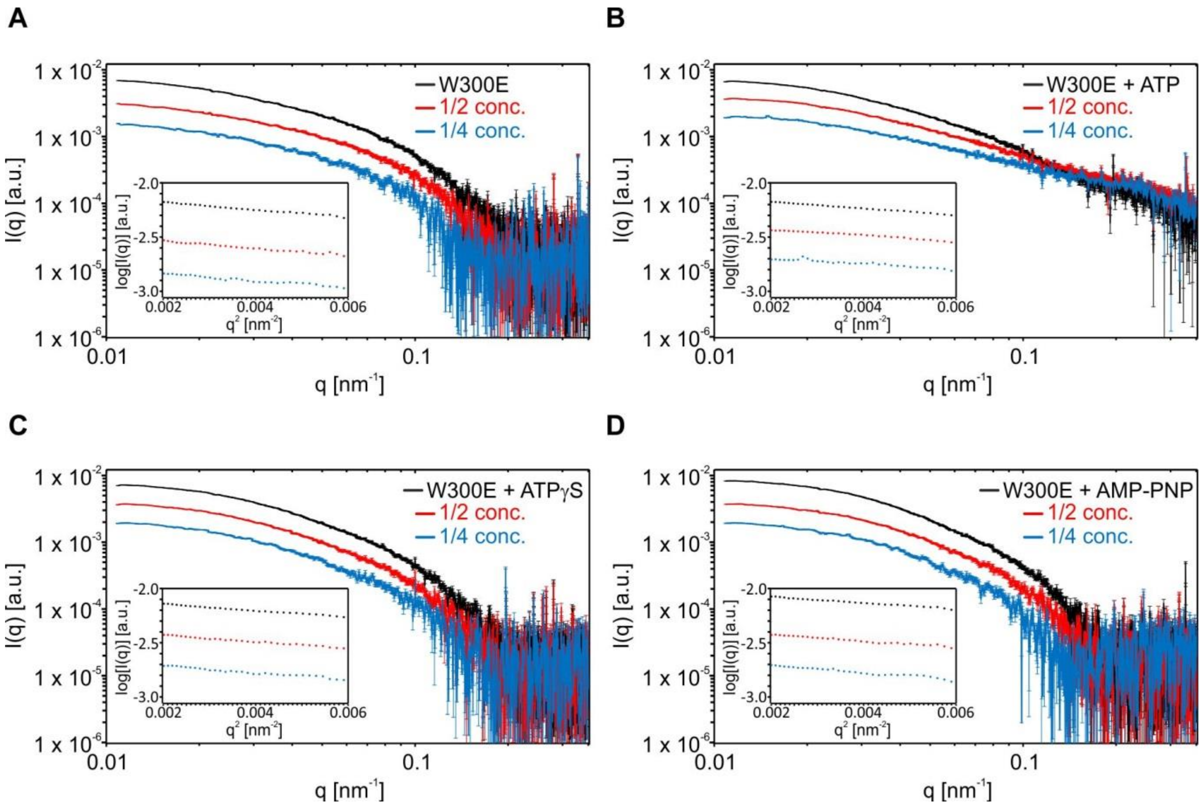

Supplementary Figure 7: SAXS scattering curves. Experimental X-ray scattering data of Hsp82 W300E, free (A) and in presence of ATP (B), ATPγS (C), or AMP-PNP (D) recorded at different sample concentrations in the range 1-5 mg/ml. Both the *q*, and *I(q)* axes are shown in a logarithmic representation. The angular ranges from 0.0012 - 0.4 nm^{-1} are compared.

Supplementary Figure 8

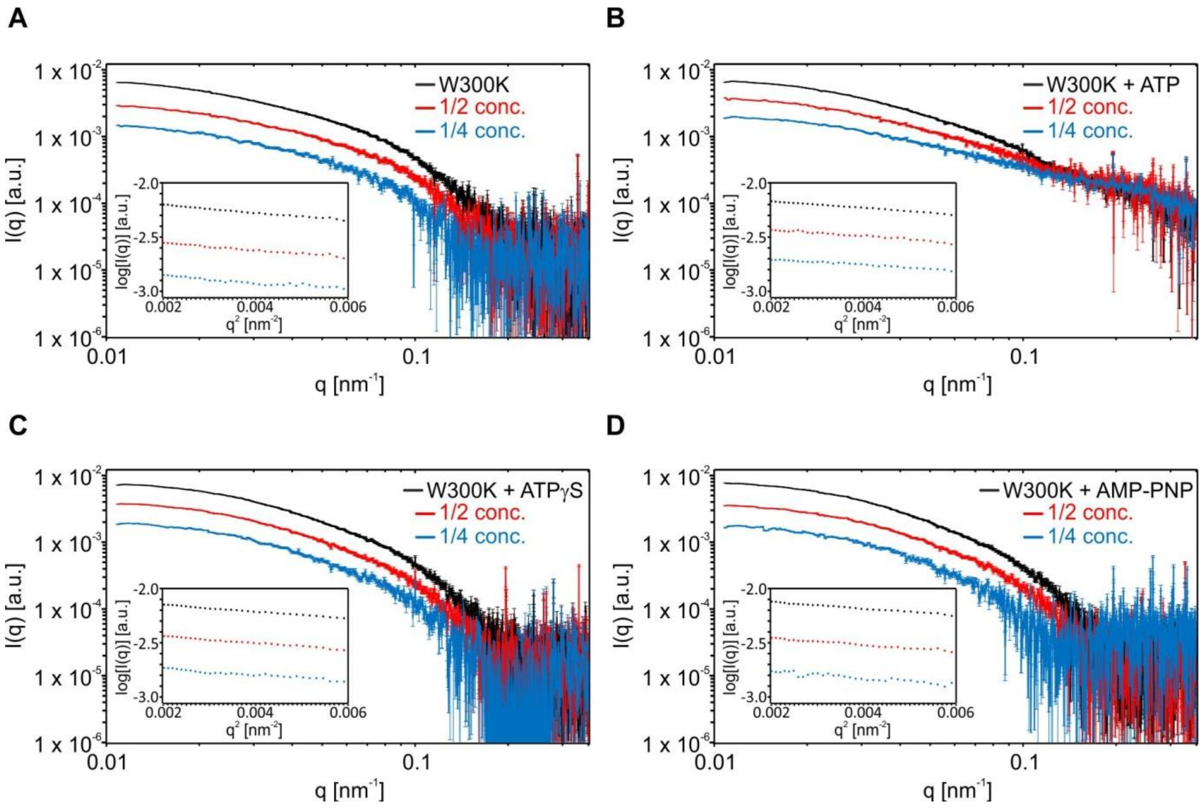

Supplementary Figure 8: SAXS scattering curves. Experimental X-ray scattering data of Hsp82 W300K, free (A) and in presence of ATP (B), ATPγS (C), or AMP-PNP (D) recorded at different sample concentrations in the range 1-5 mg/ml. Both the *q*, and *I(q)* axes are shown in a logarithmic representation. The angular ranges from 0.0012 - 0.4 nm^{-1} are compared.

Supplementary Figure 9

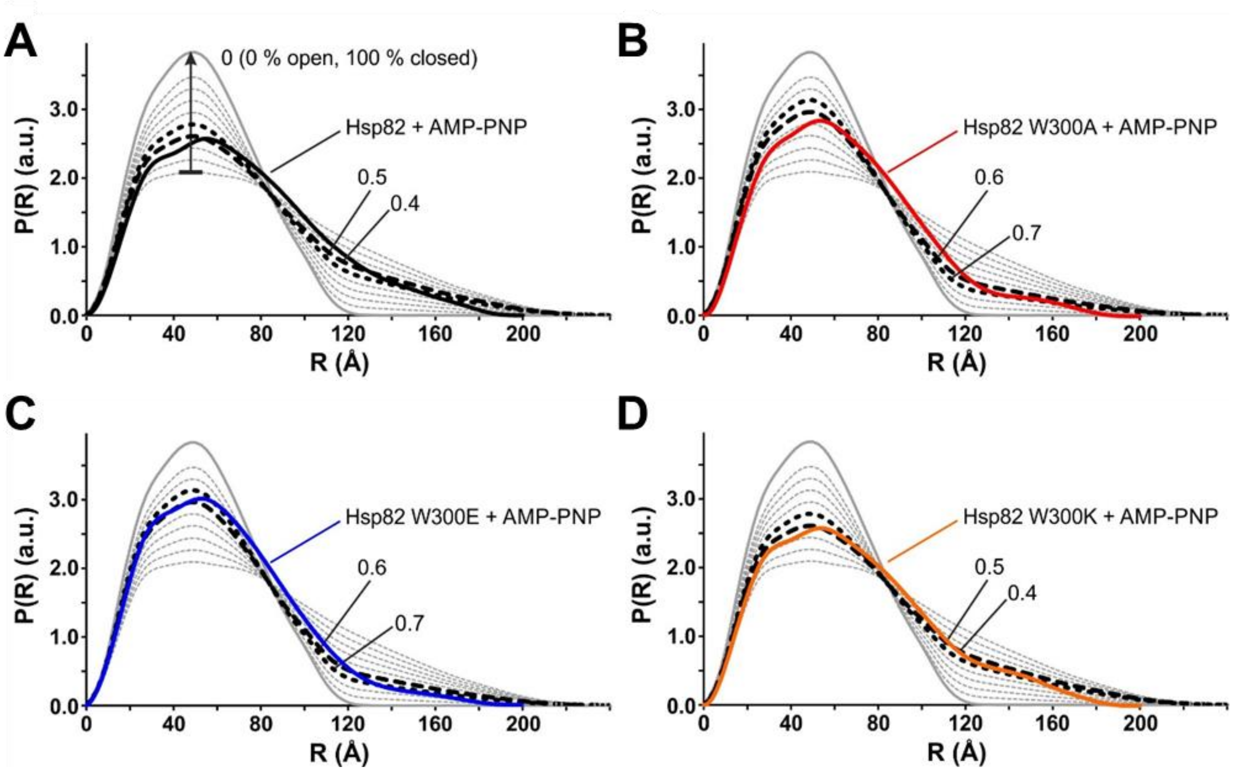

Supplementary Figure 9: Comparison of experimental radial density distribution of (A) wt Hsp90, (B) Hsp90 W300A, (C) Hsp90 W300E and (D) Hsp90 W300K with theoretical radial density distributions calculated for a mixture of open and closed complexes in presence of AMP-PNP. For population analysis the radial density distribution of open Hsp90 (experimental) and of closed Hsp90 (PDB ID 2CG9, back-calculated using the program Crysol) were taken as input.

Supplementary Figure 10

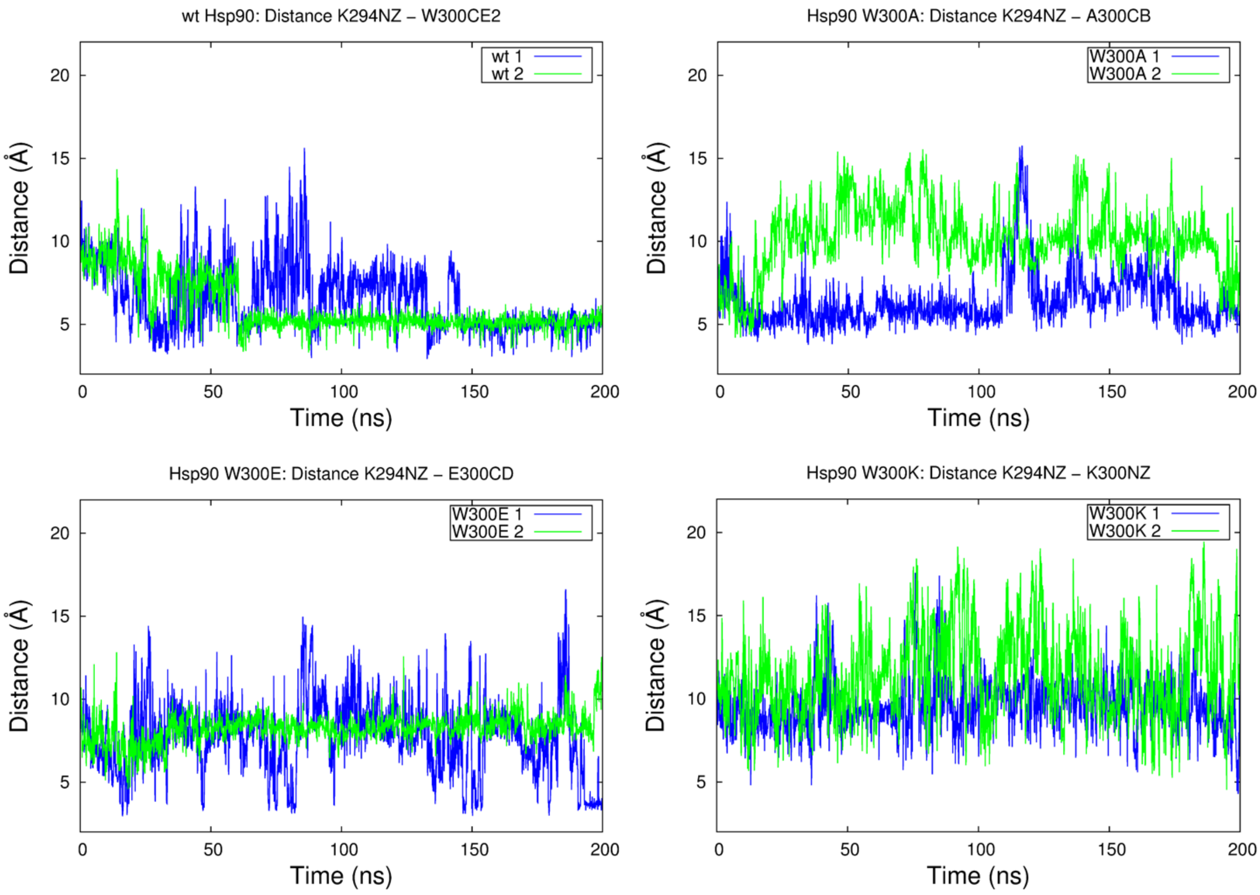

Supplementary Figure 10: Distance between residue 300 and Lys-294 in the MD simulations. The cation-pi interaction between W300 and K294 can be seen as a *ca*. 5 Å distance between the residues.

Supplementary Figure 11

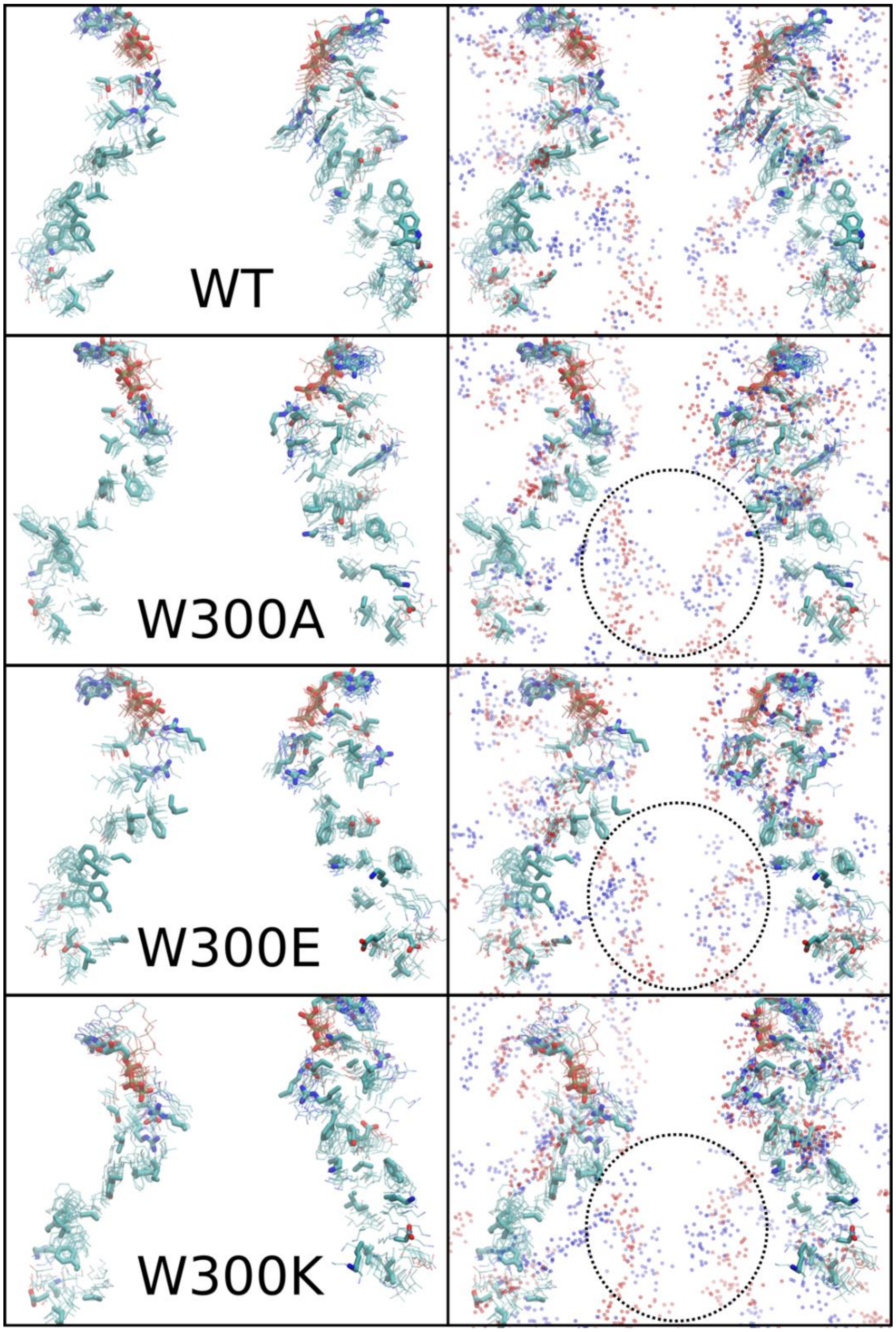

Supplementary Figure 11: Dynamic networks. *Left:* Dynamics of the putative commutation network between W300 and the ATP-binding site in WT Hsp90 and W300A, W300E, W300K mutants during 200 ns MD simulations. The figure shows average residue positions. *Right*:

dynamics of charged residues, with the Hsp90 interface. Circled areas show an increase in dimer interactions.

Supplementary Figure 12

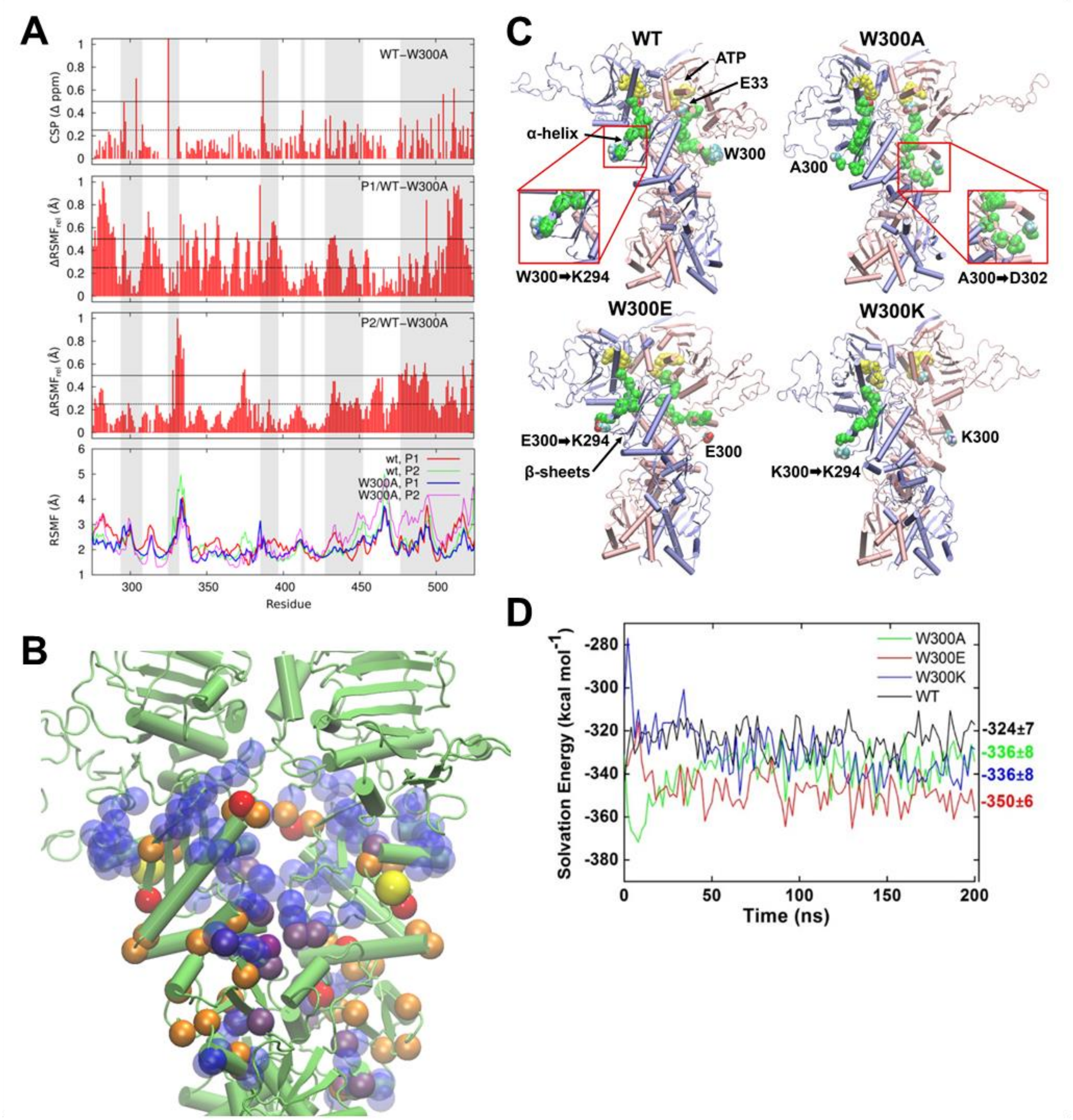

Supplementary Figure 12: MD simulation data and force propagation pathways in wt and mutant Hsp90; (A) Comparison of chemical shift perturbations (CSP) between *wt* and W300A (top), with root-mean-square-fluctuations (RMSF) obtained from 200 ns MD simulations of the *wt* and W300A-mutated Hsp90 (bottom) for the two chains of Hsp90 (P1 and P2). The two middle panels show RMSF differences between *wt* and W300A for the two Hsp90 domains, here P1 and P2, respectively. Gray shaded areas mark residues with *medium*-to-*large* CSPs. (B) Comparison of residues that have high RMSF differences between the *wt* and W300A; MD

simulations (in transparent blue) with residues that undergo medium (in orange) to large (in red) chemical shift perturbation (CSP) upon the W300A substitution. The overlapping residues are show in purple. W300 is shown as a yellow sphere. (C) Global structures of the wild type and W300A, W300E, W300K mutants of Hsp90, obtained after 200 ns MD simulations, and force propagation pathways from W/A/E/K300 to E33, marked in green with van-der-Waals representation. The two monomers of Hsp90 are shown in blue and pink, and ATP is shown in yellow. (D) Solvation free energy of the ATP-Mg^{2+} complex in Hsp90 obtained from Poisson-Boltzmann continuum electrostatics calculations.

Supplementary Figure 13

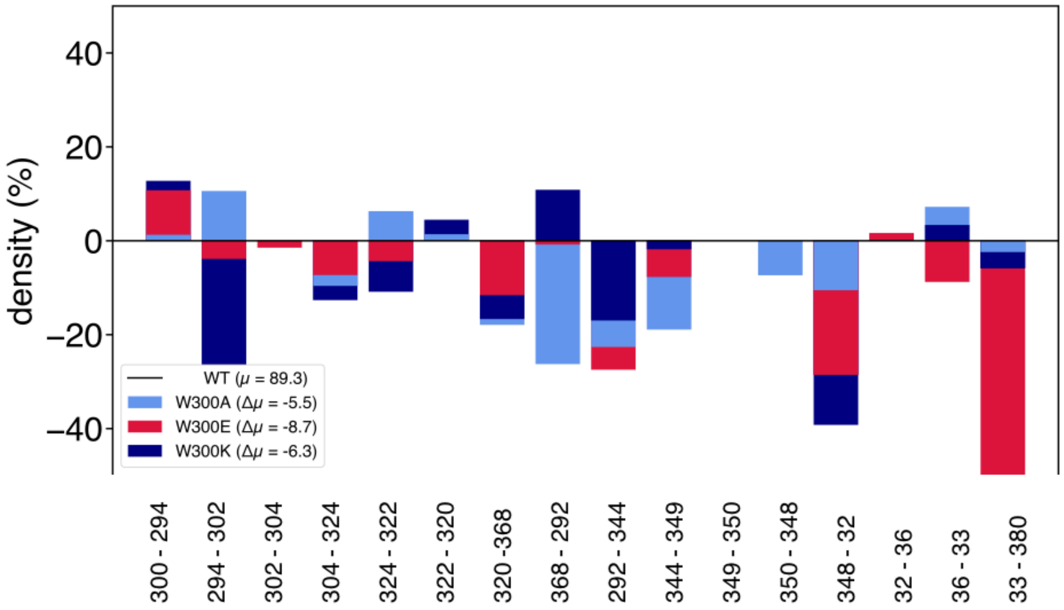

Supplementary Figure 13: Protein packing analysis. Analysis of changes in pair-wise protein packing densities between the W300-mutants and wild type Hsp90 (density-%) along the putative interactions networks between residue 300 and the ATP-binding site for wt-W300A (light blue), wt - W300E (red), wt - W300K (dark blue), computed as defined in SI Ref. 2 [1]. The average changes in density (Δμ) along the path are given in the legend.

Supplementary Figure 14

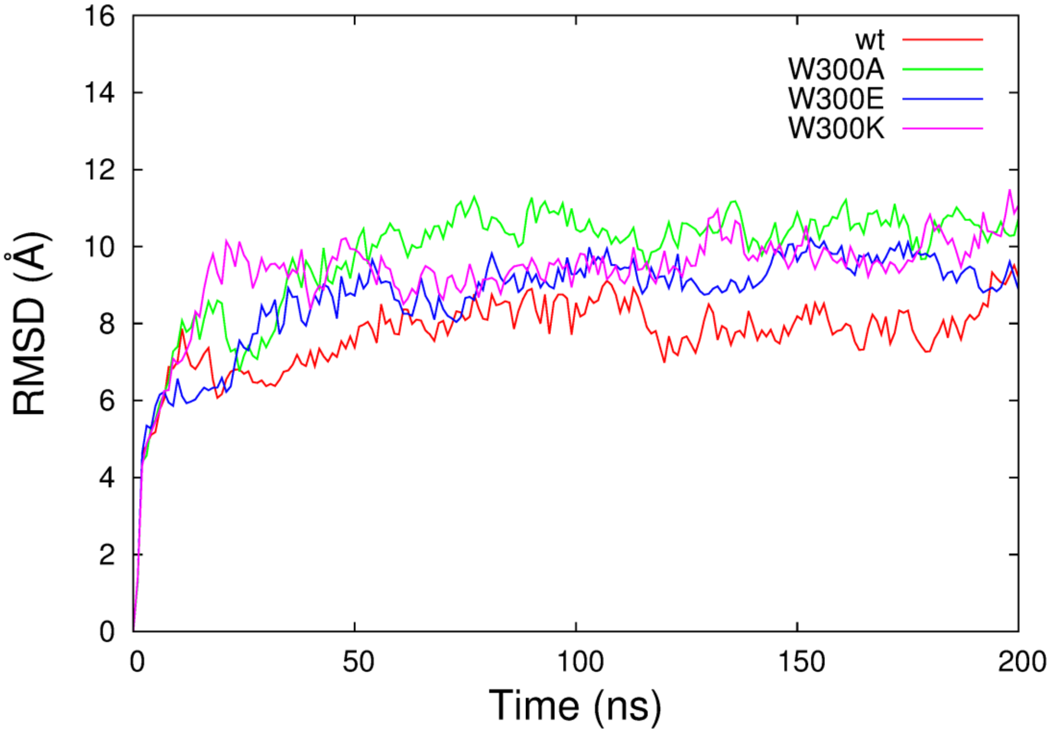

Supplementary Figure 14: Protein backbone analysis. Backbone root-mean-square-deviation (RMSD) of the *wt*, and *in silico*-mutated W300A, W300E, and W300K models of Hsp90 as a function of the simulation time.

Supplementary Tables

Supplementary Table 1

Supplementary Table 1: The W300 mutation affects several residues *in vitro* and *in* silico

Intermediate CSP (>0.25 ppm)	Strong CSP (>0.5 ppm)	Intensity change (<0.34)	RMSF	RMSF	CSP and RMSF (strong CSP in red)	Intensity change and RMSF
I296	L304	Q286	W277	I396	I296	Q286
S297	K325	L304	R279	E431	L304	S310
H308	K387	V306	N280	D432	L331	V311
L331	I505	K307	P281	T433	F332	E312
F332	Q512	H308	S282	Q434	E431	Y344
N386		S310	D283	G476	E477	F349
I388		V311	I284	E477	K480	L374
E412		E312	T285	S478	T511	I392
Q413		E316	Q286	K480	Q512	R393
G428		R326	E287	A481	L513	G476
E431		Y344	E288	P486	G518	F516
R436		V345	Y289	F487	I524	
A440		R346	N290	K492		
K441		R347	I296	A493		
K449		F349	S310	K494		
L454		I350	V311	Y508		
E477		E353	E312	A509		
K480		F364	G313	F510		
S485		G367	Q315	T511		
L488		S371	L328	Q512		
E497		E372	D330	L513		
T502		L374	L331	K514		
T511		L376	F332	E515		
L513		I392	E333	F516		
L521		R393	S334	E517		
G518		K399	K335	G518		
I524		G428	L343	I524		
		H430	Y344			
		R444	F349			
		N446	D356			
		S447	L357			
		T448	I358			
		L454	V369			
		L457	D370			
		D459	L374			
		T475	P375			
		G476	N377			
		V482	Q385			
		E497	I392			
		F516	R393			
		D527	K394			
			N395			

List of residues that undergo chemical shift perturbations (CSP), intensity changes, *root-mean-square-fluctuations* (RMSF) or a combination of both upon the W300A mutation in Hsp90-M. Residues that undergo strong CSP and RMSF are shown in red.

Supplementary Table 2

Supplementary Table 2: SAXS data collection and analysis

Data-collection parameters	
Instrument	SAXSess mc^2 (Anton Paar)
Beam geometry	10 mm slit
Wavelength (Å)	1.5418
q range (nm^{-1})	0.012 - 0.63
Exposure time (min)	90
Concentration range (mg/ml)	1–5
Temperature (K)	298
Software employed	
Primary data reduction	SAXSquant (version 3.9)
Data processing	GIFT, GNOM

Supplementary Table 3

Supplementary Table 3: SAXS data and analysis

Sample	R_g [Å]	D_{max} [Å]	Molecular mass [kDa]*
Hsp82	67.0 ± 0.2	240	183
Hsp82 W300A	64.6 ± 0.3	240	171
Hsp82 W300E	65.9 ± 0.3	240	169
Hsp82 W300K	67.7 ± 0.2	260	178
Hsp82 + ATP	72.2 ± 0.4	300	200
Hsp82 W300A + ATP	65.8 ± 0.3	240	162
Hsp82 W300E + ATP	67.6 ± 0.2	240	182
Hsp82 W300K + ATP	72.3 ± 0.5	300	200
Hsp82 + ATPγS	58.5 ± 0.2	230	186
Hsp82 W300A + ATPγS	53.7 ± 0.3	190	177
Hsp82 W300E + ATPγS	55.0 ± 0.2	230	190
Hsp82 W300K + ATPγS	62.0 ± 0.2	230	200
Hsp82 + AMP-PNP	55.6 ± 0.2	200	193
Hsp82 W300A + AMP-PNP	51.8 ± 0.1	200	176
Hsp82 W300E + AMP-PNP	49.7 ± 0.2	200	165
Hsp82 W300K + AMP-PNP	55.0 ± 0.2	200	175

*The molecular mass was determined using Porod's law.

Supplementary Table 4

Supplementary Table 4: Primer sequences

Mutant Primer	Sequence
W300A foward	CTATAAGTCTATTTCAAACGACGCGGAAGACCCATTGTACGTTAAG
W300A reverse	CTTAACGTACAATGGGTCTTCCGCGTCGTTTGAAATAGACTTATAG
W300E forward	CTATAAGTCTATTTCAAACGACGAAGAAGACCCATTGTACGTTAAG
W300E reverse	CTTAACGTACAATGGGTCTTCTTCGTCGTTTGAAATAGACTTATAG
W300K forward	CTATAAGTCTATTTCAAACGACAAAGAAGACCCATTGTACGTTAAG
W300K reverse	CTTAACGTACAATGGGTCTTCTTTGTCGTTTGAAATAGACTTATAG
W300F forward	TTCAAACGACTTTGAAGACCCATTG
W300F reverse	ATAGACTTATAGAAAGCATTGTATTC
W300Y forward	TTCAAACGACTATGAAGACCCATTG
W300Y reverse	ATAGACTTATAGAAAGCATTGTATTC
W277A forward	ATAAGCCTTTGGCGACTAGAAACCC
W277A reverse	GTCTTGTTTAGTTCTTCTATCTC
W312A forward	CACTAATGACGCCGAAGACCACTTGGC
W312A reverse	AGGCTCTTGTAGAATTCTC
W312E forward	CACTAATGACGAAGAAGACCACTTGGC
W312E reverse	AGGCTCTTGTAGAATTCTC
W312K forward	CACTAATGACAAAGAAGACCACTTGGC
W312K reverse	AGGCTCTTGTAGAATTCTC

Supplementary Methods

Expression and purification of human GR-LBD, Hsp90/-mutants, human Hsp90β/-mutants, Hsp40 (Ydj-1), Hsp70, Hop and p23

Human GR-LBD (aa 527-777, F602S/A605V/V702A/E705G/M752T) was expressed at 18°C overnight in ZYM-5052 media supplied with 500 µM Dexamethasone (DEX) (Sigma-Aldrich, St. Louis, USA). Expression of human apo-GR-LBD was performed in hormone-free ZYM-5052 media at 16°C overnight. Cells were harvested by centrifugation for 15 min at 7,000 rpm and 4°C (Beckman Avanti J-26 XP, Beckman Coulter, Brea, California) and washed with ice-cold PBS. Cells were resuspended in Ni-A buffer (50 mM Tris, 2 M Urea, 100 mM NaCl, 5 mM $MgCl_2$, 10 mM Imidazole, 2 mM ß-mercaptoethanol, 50 µM DEX, pH 7.9) supplemented with DNaseI (Roche, Basel, Swiss) and Protease Inhibitor HP (Serva electrophoresis GmbH, Heidelberg, Germany). Cell suspension was lysed by sonication (Bandelin Sonoplus UW2200, Bandelin electronic, Berlin Germany) or french press (Constant Systems Limited, Low March, UK) and centrifuged for 1 hour at 20,000 rpm and 4°C. Cleared lysate was applied onto a Ni-column (5 ml FF,GE Healthcare, Chalfont St. Giles, Great Britain), pre-equilibrated in Ni-B buffer (50 mM Tris, 500 mM NaCl, 10 mM Imidazole, 10% Glycerol, 2 mM ß-mercaptoethanol, 50 µM DEX pH 7.9). The column was then gradient-equilibrated in Ni-B buffer and His_6-Halo-GR-LBD was eluted with Ni-C buffer (50 mM Tris, 500 mM NaCl, 350 mM Imidazole, 10% Glycerol, 2 mM ß-mercaptoethanol, 50 µM DEX pH 7.9). IMAC-Buffers for the purification of apo-GR-LBD were supplied with 2 mM ATP to prevent binding of *E. coli* GroE and DnaK. GR-protein containing fractions were pooled, supplemented with His_6-tagged TEV protease and dialyzed against 50 mM Tris, 100 mM NaCl, 10% Glycerol, 2 mM ß-mercaptoethanol, 0.5% CHAPS, 50 µM DEX pH 7.9 overnight. Digested protein was passed through a Ni-column to remove Halo-tag-Fusion and TEV protease. The flow through was concentrated and loaded onto a gelfiltration column (Superdex 200, 16/60 pg, GE Healthcare, Chalfont St. Giles, Great Britain) equilibrated in GR-storage buffer (25 mM Tris, 100 mM NaCl, 10% Glycerol, 2 mM DTT, 50 µM DEX pH 7.9). GR-proteins were shock-frozen and analyzed by SDS-PAGE.

Hsp90/-mutants and human Hsp90β/-mutants constructs were expressed at 37°C for 4 h with 1 mM IPTG. Cells were harvested by centrifugation for 20 min at 6,000 rpm and 4°C (Beckman Avanti J-26 XP, Beckman Coulter, Brea, California) and resuspended in Ni-A buffer (40 mM KPi, 150 mM KCl, 6 mM Imidazol, pH 7.5) supplemented with DNaseI (Roche, Basel, Swiss) and Protease Inhibitor HP (Serva electrophoresis GmbH, Heidelberg, Germany). Cell suspension was lysed by french press (Constant Systems Limited, Low March, UK) and centrifuged for 35

min at 20,000 rpm and 4°C. The cleared lysate was loaded on a Ni-column (5 ml FF,GE Healthcare, Chalfont St. Giles, Great Britain) and eluted with 300 mM imidazole. Fractions containing the target protein were pooled and diluted to 150 ml with ResQA buffer (40 mM Hepes, 20 mM KCl, 1 mM EDTA, 1mM DTT pH 7.5) to dilute the salt concentration that would prevent the protein from binding the anion-exchange column. The protein was then loaded on the ResQ column (6 ml, GE Healthcare, Chalfont St. Giles, Great Britain) equilibrated in ResQA buffer. After loading, the column was washed with 10 CV ResQA buffer and eluted using a gradient from 0-50% ResQB buffer (40 mM Hepes, 1 M KCl, 1 mM EDTA, 1mM DTT pH7.5). Protein-containing fractions were pooled, concentrated and loaded on a Superdex 16/60 200 pg gel-filtration column (GE Healthcare, Chalfont St. Giles, Great Britain). Purity of the protein charge was controlled by SDS-PAGE and the protein was frozen in liquid nitrogen.

Human Hsp70 and yeast Ydj-1 (Hsp40) were expressed as SUMO-protein fusion constructs. Therefore anion-exchange chromatography was omitted. Instead, after proteolytic digest overnight by His_6-tagged SUMO-protease, the proteins were loaded again on a Ni-A equilibrated HisTrap FF column. During SUMO-cleavage also the His_6-tag was cleaved off, so the target proteins eluted during the flow trough. The flow through was then concentrated and loaded on a Superdex 16/60 200 pg gel filtration column. To prevent binding of DnaK form *E. coli* 0.5 mM ATP were added to Ni-A and Ni-B buffers.

Human co-chaperones Hop and p23 were expressed tag-free in *E. coli.* Purification was conducted via a combination of anion-exchange, hydroxyapatite (HAT) and gel-filtration chromatography. Cells were harvested, disrupted and the lysate cleared as mentioned for the other proteins. The lysate was loaded on a self-packed Q-Sepharose column (GE Healthcare, Chalfont St. Giles, Great Britain) and eluted via a linear gradient form 0-600 mM KCl. The fractions containing the target protein were diluted to 150 ml in HAT low-salt buffer (10 mM KPi, pH 7.0) and subsequently applied to the self-packed HAT-column (GE Healthcare, Chalfont St. Giles, Great Britain). Protein elution was reached by applying a linear gradient of 0-100% HAT high-salt buffer (400 mM KPi, pH 7.0). Desired protein fractions were combined and loaded on a Superdex 16/60 75 or 200 pg gel-filtration column (GE Healthcare, Chalfont St. Giles, Great Britain). Protein purity was assessed by SDS-PAGE analysis.

Fluorescence-anisotropy based hormone rebinding assay

GR-LBD ligand binding recovery was assessed as reported by Kirschke and co-workers [2]. Experiments were conducted on a Jasco Fluorescence Spectrometer FP-8500 equipped with polarizers (Jasco, Groß-Umstadt, Germany). Measurements were performed in 30 mM HEPES, 150 mM KCl, 5 mM $MgCl_2$, pH 7.5 and 5 mM ATP. 1 µM apo-GRLBD was pre-incubated with 2 µM Hsp40, 15 µM Hsp70 and 5 mM ATP for 60 min at room temperature, followed by incubation with 50 nM F-DEX for 60 min. Addition of 15 µM Hop, 15 µM p23 and 15 µM human Hsp90β/-mutants initiated hormone-rebinding.

Yeast cell lysis and Western Blotting

After 5-FOA shuffling, single clones were grown at 30 °C in minimal medium (5 ml) to stationary phase. After an OD_{600} of 5 was reached, cells were harvested (4500 rpm., 5 min). The cell pellets were washed with H_2O, and the OD_{600} was measured again. Cells were spun down (4500 rpm, 5 min) and resuspended in 200 µl H_2O; then 200 µl of 0.2 M NaOH was added, and cells were incubated for 3 min at RT. Cells were harvested again (14000 rpm., 1 min), and supernatant was discarded. The pellet was resuspended in 100 µl (OD_{600} of 5) sample buffer and boiled for 5 min at 95 °C. Proteins were separated by SDS–PAGE and blotted on a PVDF membrane for 1 h. The following primary antibodies were used to probe the membrane for 1 h at RT: anti-yeast Hsp90 (Pineda Antibody Service, Berlin, Germany), polyclonal, produced in rabbit (1:20000 dilution in TBS 0.1% Tween 1% milk) and anti-PGK1 (Invitrogen, Carlsbad, USA), monoclonal, produced in mouse (1:20000 in PBS 0.1% Tween 1% milk). The secondary antibodies were anti-mouse and anti-rabbit IgG-peroxidase antibodies (Sigma-Aldrich, St. Louis, USA), diluted 1:20,000 in PBS 0.1% Tween 1% milk, were used to detect the respective first antibody for 45 min at RT. For all antibodies, validation is provided on the manufacturers' websites. Detection was performed with Western Bright ECL Spray (Advansta, Menlo Park, USA) and ImageQuant LAS4000 (GE Healthcare, Chalfont St. Giles, Great Britain).

Supplementary References

1. Kaila, V.R., Johansson, M.P., Sundholm, D. & Wikstrom, M. Interheme electron tunneling in cytochrome c oxidase. *Proc Natl Acad Sci U S A* **107**, 21470-5 (2010).
2. Kirschke, E., Goswami, D., Southworth, D., Griffin, P.R. & Agard, D.A. Glucocorticoid receptor function regulated by coordinated action of the Hsp90 and Hsp70 chaperone cycles. *Cell* **157**, 1685-97 (2014).
